# Intestinal Alkaline Phosphatase Exerts Anti-Inflammatory Effects Against Lipopolysaccharide by Inducing Autophagy

**DOI:** 10.1038/s41598-020-59474-6

**Published:** 2020-02-20

**Authors:** Sudha B. Singh, Amanda Carroll-Portillo, Cristina Coffman, Nathaniel L. Ritz, Henry C. Lin

**Affiliations:** 1 Biomedical Research Institute of New Mexico, VA Health Care System, Albuquerque, New Mexico USA 87108; 2 Section of Gastroenterology, Medicine Service, New Mexico VA Health Care System, Albuquerque, New Mexico USA 87108; 3 Division of Gastroenterology and Hepatology, Department of Medicine, the University of New M5052651711exico, Albuquerque, New Mexico 87131 USA; 4 Present Address: Department of Anatomy & Neuroscience, University College Cork; APC Microbiome institute, University College Cork, Cork, Ireland

**Keywords:** Macroautophagy, Monocytes and macrophages

## Abstract

Intestinal alkaline phosphatase (IAP) regulates bicarbonate secretion, detoxifies lipopolysaccharide (LPS), regulates gut microbes, and dephosphorylates proinflammatory nucleotides. IAP also exhibits anti-inflammatory effects in a Toll-like Receptor-4 (TLR-4) dependent manner. However, it is not known whether IAP induces autophagy. We tested the hypothesis that IAP may induce autophagy which may mediate the anti-inflammatory effects of IAP. We found that exogenous IAP induced autophagy in intestinal epithelial cells and in macrophages. TLR4INC34 (C34), a TLR4 signaling inhibitor, suppressed IAP-induced autophagy. IAP also inhibited LPS-induced IL-1β mRNA expression and activation of NF-κB. When autophagy was blocked by 3-methyladenine (3MA) or by *Atg5* siRNA, IAP failed to block LPS-mediated effects. IAP also upregulated autophagy-related gene expression in small intestine in mice. We administered either vehicle or IAP (100 U/ml) in drinking water for 14 days in C57BL/6 mice. Mice were sacrificed and ileal tissues collected. Increased expression of *Atg5*, *Atg16*, *Irgm1*,* Tlr4*, and *Lyz* genes was observed in the IAP treated group compared to the vehicle treated group. Increase in Atg16 protein expression and fluorescence intensity of LC3 was also observed in IAP-treated tissues compared to the vehicle-treated tissues. Thus, our study lays the framework for investigating how IAP and autophagy may act together to control inflammatory conditions.

## Introduction

Intestinal alkaline phosphatase (IAP) belongs to the family of alkaline phosphatases that are classified into four categories in humans. These include tissue non-specific alkaline phosphatase (TNAP), placental alkaline phosphatase (PLAP), germ cell alkaline phosphatase (GCALP), and intestinal alkaline phosphatase (IAP). IAP, a brush border enzyme, is produced by intestinal epithelial cells and is secreted in the intestinal lumen, blood, and in stool. IAP plays a critical role in maintaining gut homeostasis by functions such as detoxification of lipopolysaccharide (LPS)^1^, dephosphorylation of proinflammatory nucleotides^2^, regulation of bicarbonate secretion and duodenal surface pH^3^, absorption of intestinal long-chain fatty acids (LCFA), and regulation of gut microbiome^4^. IAP protects against systemic infections and inflammatory diseases^1,5,6^. A decreased expression of IAP is found in disorders such as inflammatory bowel disease (IBD)^7,8^, metabolic syndrome^9^, cystic fibrosis^10^, necrotizing enterocolitis^11^, and diabetes^12^. Treatment with exogenous IAP was found to improve inflammation and gut barrier function in mouse models of these diseases^11,13,14^. However, the detailed underlying mechanisms of how IAP deficiency is linked to these diseases and the downstream protective mechanisms of exogenously administered IAP remain largely unexplored. A recent study demonstrated that IAP inhibited LPS-induced TNFα and IL-6 proinflammatory cytokine production, NF-κB DNA-binding activity, and IκBα phosphorylation /degradation in macrophages derived from wild type mice but not in macrophages derived from Toll-like receptor-4 (TLR4)-/- mice^15^. Moreover, IAP reduced the severity of DSS-induced colitis in mice in a TLR4-dependent manner. It is known that TLR4 signaling acts as a sensor for autophagy^16–18^. Autophagy is a cellular degradative pathway that protects against intestinal pathogens and inflammation, among its other vital functions^19^. Autophagy begins with the formation of a double membrane structure called phagophore which engulfs the cargo to be degraded to become a double membrane autophagosome. The autophagosome then fuses with the lysosome to become an autolysosome where the cargo is degraded by the activity of lysosomal enzymes^20^. The complete process of degradative activity of autophagy is referred to as autophagy flux. Polymorphism in autophagy genes ATG5, ATG16L1, and IRGM have been linked to Crohn’s disease(CD)^21^, necrotizing enterocolitis^22^, and sepsis^23^, conditions that are interestingly also linked to a decreased expression of IAP. Based on these observations, we tested the hypothesis that IAP may induce autophagy in a TLR4- dependent manner. We also investigated whether IAP inhibited pro-inflammatory effects of LPS in an autophagy-dependent manner.

## Results

### IAP induces autophagy in HCT116 epithelial cells

**Figure 1:**
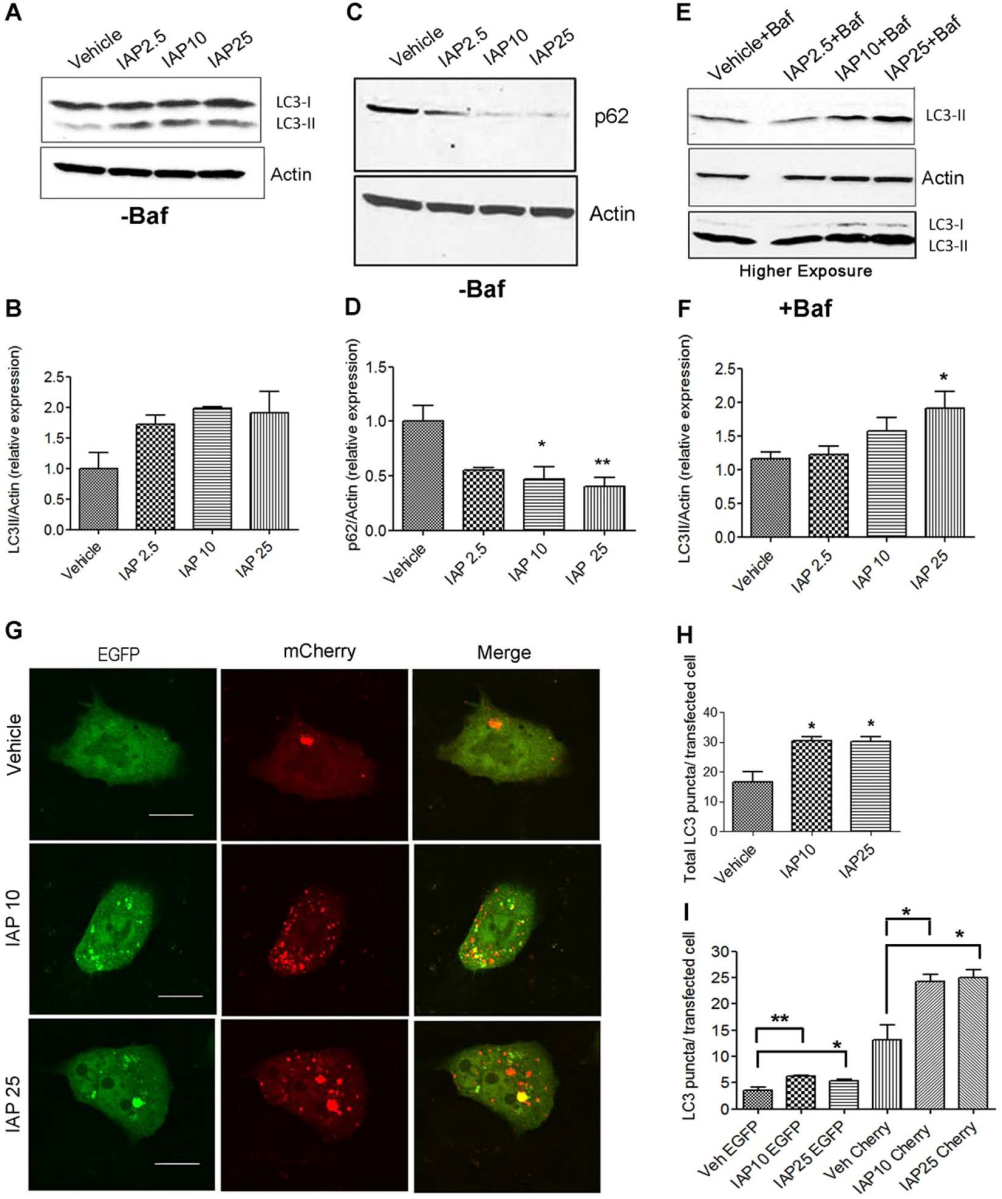
IAP induces autophagy in HCT116 epithelial cells. (**A**) HCT116 cells were treated with 2.5, 10, or 25 U/ml of bovine IAP (A2356) for 24 hrs. Vehicle-treated cells were used as controls. Fifty µg of protein lysate was separated on SDS page and analyzed for LC3-II induction by Western blotting. Actin was used as a loading control. Representative image indicates the gel that was cut at 25 kDa and lower portion was probed for LC3 while upper portion was probed for actin. (**B)** Blots were quantified with ImageJ by analyzing the ratio of LC3-II/actin (mean ± SEM from three independent experiments). (**C**) Cells treated with IAP same as in A and probed for p62. The membranes were cut at 100 kDa and 25 kDa. Following p62 detection, the membranes were stripped and probed for actin. (**D)** Blots were quantified with ImageJ by analyzing the ratio of p62/actin (mean ± SEM from three independent experiments). (**E**) Cells treated with IAP same as in A, but in the presence of bafilomycin A1 (100 nM for 3 h towards the end of 24 hr period). LC3-II induction was analyzed by Western blotting and (**F**) quantified with ImageJ (mean ± SEM from three independent experiments). Blot represents a gel that was cut between 75 kDa (probed for actin ~37 kDa) and at 25 kDa (probed for LC3 at ~16 &14 kDa). LC3-I was detected only after higher exposure. (**G**) Cells were transfected with mCherry-EGFP-LC3B expressing plasmid for 24 hours using lipofectamine 2000. Cells were then treated with IAP for 24 hours and fixed with 4% paraformaldehyde. After washing with PBS, cells were mounted with permafluor mounting medium (ThermoFisher), observed, and imaged with the Olympus FluoView FV1200 confocal microscope. Scale bar = 10 µm. (**H&I**)Total number of LC3 puncta per transfected cell and mCherry^+^ EGFP^+^ (yellow) and mCherry^+^ EGFP^−^ (red) puncta per transfected cell, representing autophagosomes and autolysosome respectively, were counted and plotted. One way ANOVA and Dunnett’s Multiple Comparison Test was used to determine statistical significance. *P < 0.05 and **P < 0.01, compared to vehicle.

We first tested the effect of IAP on autophagy in human intestinal cell line HCT116. Cells were treated with either IAP (2.5, 10 or 25 U/ml) or with equal volume of vehicle for 24 hours and analyzed for LC3-II induction, a gold standard for monitoring autophagy. We found that IAP increased LC3-II expression in HCT116 (Fig. 1A,B). We also observed a significant decrease in the expression of p62 (Fig. 1C), a selective substrate degraded by autophagy, at 10 U/ml (vehicle: 1.00 ± 0.14 vs IAP: 0.46 ± 0.11, p < 0.05) and at 25 U/ml (0.40 ± 0.08, p < 0.01) (Fig. 1D). As increase in LC3-II expression may indicate either autophagy activation or a block in autophagy flux^24^, we used bafilomycin A1 to monitor autophagic flux. Bafilomycin A1 (Baf) prevents the fusion of autophagosomes with autolysosomes and prevents LC3-II degradation. Thus, accumulation of LC3-II in the presence of Baf would reflect the amount of LC3-II that would have been degraded by autophagy as a result of autophagic flux. LC3-II expression increased in response to IAP in the presence of Baf with a significant difference observed at 25 U/ml (vehicle: 1.15 ± 0.10 vs. IAP: 1.91 ± 0.24, p < 0.05) suggesting that IAP induced autophagic flux (Fig. 1E,F). Also, we observed that in Baf treated cells, the LC3-II band intensity was higher than LC3-I and LC3-I was detected only after a prolonged exposure (Fig. 1E). Thus, Western blots of Baf treated cells mainly showed one band representing LC3-II which corresponds to ~14 kDa. We further monitored autophagy flux by transfecting the cells with tandem labeled mCherry-EGFP-LC3B plasmid (Fig. 1G-I). As EGFP is degraded by the acid proteases in the lysosomes, mCherry^+^ EGFP^+^ puncta represent only autophagosomes. In contrast, mCherry^+^ EGFP^−^ puncta are acid-insensitive and represent autolysosomes. Thus, increase in mCherry^+^ EGFP^−^ LC3 puncta in the presence of IAP would indicate autophagic flux. Transiently transfected cells were treated with either vehicle or IAP (10 and 25 U/ml) and autophagosomes (mCherry^+^ EGFP^+^ puncta) and autolysosomes (mCherry^+^ EGFP^−^ puncta) were quantified in all the groups. Compared to the vehicle treated cells, we observed a significant increase in the total number of LC3 puncta in cells treated with IAP at 10 U/ml (vehicle: 16.80 ± 3.24 vs IAP: 30.57 ± 1.27, p < 0.05) and at 25 U/ml (30.33 ± 1.65, p < 0.01) (Fig. 1H). The effects of IAP at 10U/ ml were comparable to 25U/ml. Next, we quantified mCherry^+^ EGFP^+^ LC3 puncta (yellow) that represented autophagosomes and mCherry^+^ EGFP^−^ LC3 puncta (red) that represented autolysosomes. We found a small but significant increase in mCherry^+^ EGFP^+^ puncta in the presence of IAP (vehicle: 3.60 ± 0.55 vs IAP10: 6.25 ± 0.07, p < 0.01, and IAP25:5.32 ± 0.27) when compared to the vehicle treated cells. An increase was also observed in mCherry^+^ EGFP^−^ puncta in cells treated with IAP when compared to the vehicle treated group (vehicle:13.20 ± 2.87 vs IAP10: 24.30 ± 1.25, and IAP25: 25.01 ± 1.45, p < 0.05) (Fig. 1I). Overall, these data suggest that IAP induced autophagy in epithelial cells.

### IAP induces autophagy in RAW264.7 macrophages

**Figure 2:**
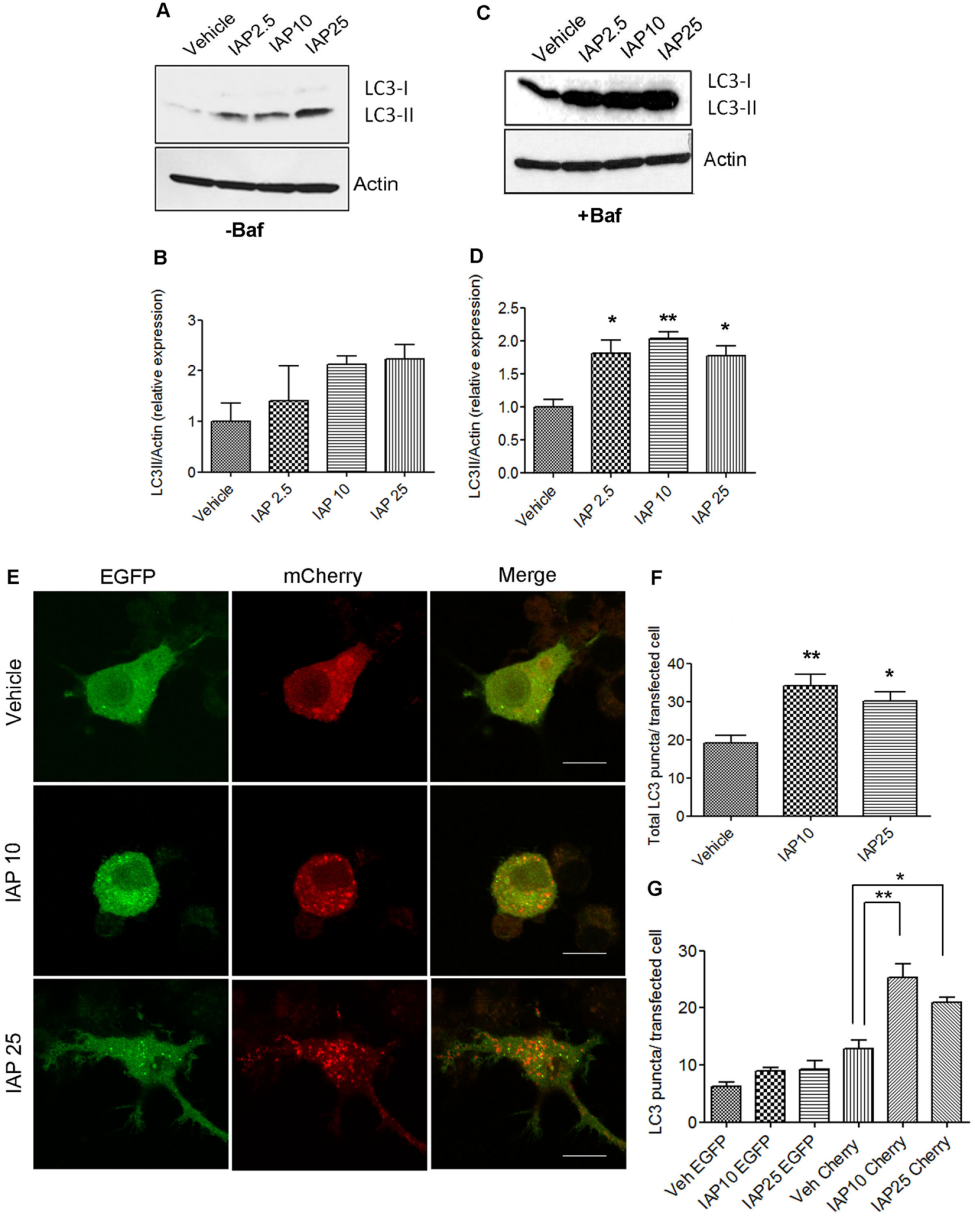
IAP induces autophagy in RAW264.7 macrophage cells. (**A**) RAW cells were treated with 2.5, 5, or 10 U/ml of bovine IAP (A2356) for 24 hrs. Vehicle-treated cells were used as controls. Fifty µg of protein lysate was separated on SDS page and analyzed for LC3-II induction by Western blotting. Actin was used as a loading control. Blot represents a gel that was cut between 75 kDa (probed for actin ~37 kDa) and at 25 kDa (probed for LC3-I at ~16 and LC3-II at ~14 kDa) (**B**) Blots were quantified with ImageJ by analyzing the ratio of LC3-II/actin (mean ± SEM from three independent experiments). (**C**) Cells were treated same as in A, but in the presence of Baf (100 nM for 3 hours towards the end). Protein samples were probed for LC3. Blot represents a gel that was cut between 75 kDa (probed for actin ~37 kDa) and at 25 kDa (probed for LC3-I at ~16 and LC3-II at ~14 kDa) (**D**) Blots were quantified with ImageJ by analyzing the ratio of LC3-II/actin (mean ± SEM from three independent experiments). (**E**) Cells were transfected with mCherry-EGFP-LC3B expressing plasmid using nucleofactor V kit. After 24 hrs, cells were then treated with IAP for 24 hours and fixed with 4% paraformaldehyde. After washing with PBS, cells were mounted with permaflour mounting medium (ThermoFisher), observed, and imaged with the Olympus FluoView FV1200 confocal microscope. Scale bar = 10 µm. (**F&G**)Total number of LC3 puncta per transfected cell and mCherry^+^ EGFP^+^ (yellow) and mCherry^+^ EGFP^−^ (red) puncta per transfected cell, representing autophagosomes and autolysosome respectively, were counted and plotted. One way ANOVA and Dunnett’s Multiple Comparison Test was used to determine statistical significance. *P < 0.05 and **P < 0.01, compared to vehicle.

Next, we examined whether IAP induced autophagy in murine RAW 264.7 macrophages (Fig. 2). While IAP is not expressed by macrophages, it is secreted into the intestinal lumen and in blood^25^ where it may function as an extracellular signaling molecule that modulates immune cells such as macrophages^26^. Similar to HCT116 cells, we found that IAP induced LC3-II accumulation in RAW cells (Fig. 2A,B). As LC3-II accumulation at a given time does not necessarily reflect autophagy flux, we measured autophagic flux in the presence of Baf in response to IAP (Fig. 2C,D). A significant increase in LC3-II expression was observed at the doses tested when compared to the vehicle control (p < 0.05). A very faint band of LC3-I was detected as compared to LC3-II. Next, cells were transfected with tandem mCherry-EGFP-LC3B plasmid and further treated with vehicle or IAP for 24 hours (Fig. 2E-G). We found that IAP increased the total number of LC3 puncta (vehicle: 19.17 ± 1.99 vs IAP10: 34.22 ± 2.83, and IAP25: 30.20 ± 2.25, p < 0.05) (Fig. 2F). While a small increase in mCherry^+^ EGFP^+^ LC3 puncta (yellow) was observed in cells treated with IAP (vehicle 6.29 ± 0.73 vs IAP10: 8.93 ± 0.59, and IAP25: 9.22 ± 1.46), a significant effect was observed in the number of mCherry^+^ EGFP^−^ LC3 puncta (red) (vehicle:12.90 ± 1.50 vs IAP10: 25.27 ± 2.38, p < 0.01 and IAP25: 20.99 ± 0.81, p < 0.05) (Fig. 2G).

**Figure 3:**
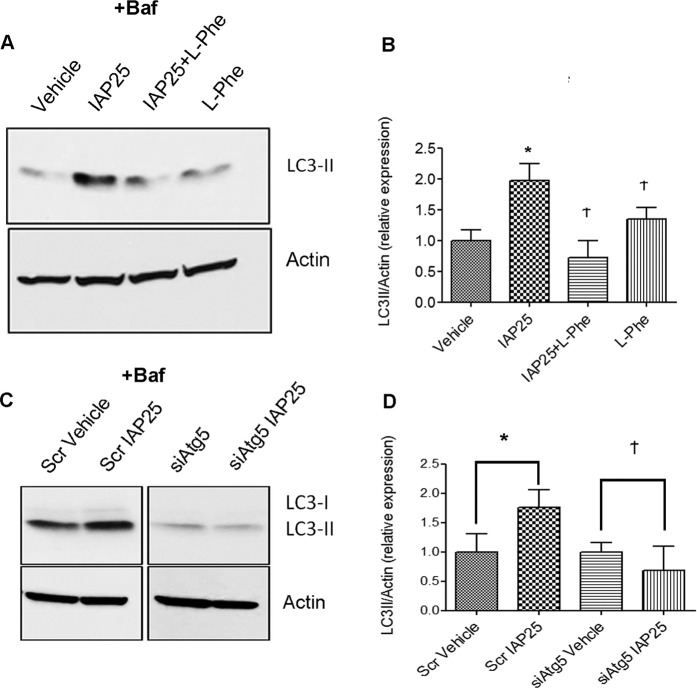
IAP-induced autophagy is inhibited by L-Phe and by *Atg5* knockdown. (**A)** RAW cells were treated with L-Phe (10 mM) or IAP alone or together for 24 hours and 100 nM Baf was added for 3 hrs towards the end. Fifty µg of protein samples were ran on SDS-PAGE and probed with LC3 and actin antibodies by Western blot. Blot represents a gel that was cut between 75 kDa (probed for actin ~37 kDa) and at 25 kDa (LC3-II at ~14 kDa) (**B**) Blots were quantified with ImageJ by analyzing the ratio of LC3-II/actin (mean ± SEM from three independent experiments). One way ANOVA and Dunnett’s Multiple Comparison Test was used to determine statistical significance. *P < 0.05 and Ϯ P > 0.05, compared to vehicle. (**C**) RAW cells were transfected with either Scr or *siAtg5* siRNA for 48 hrs. Cells were then treated with either vehicle or IAP (25U/ml) for 24 hrs and Baf was added for 3 hrs towards the end of the experiment. Cells were harvested and protein samples were subjected to Western Blotting and probed for LC3 and Actin. Blot represents two gels that were cut between 75 kDa (probed for actin ~37 kDa) and at 25 kDa (LC3-I at ~16 kDa and LC3-II at ~14 kDa) (**D**) Quantification was carried out using ImageJ and data was plotted as LC3-II/Actin ratio (relative expression, arbitrary units). Students two-tailed T-test was used to compare significance between the vehicle and IAP treatment within a group. *P < 0.05 and Ϯ P > 0.05.

To rule out the possibility that induction of LC3-II by IAP was likely due to potential contaminants such as LPS in the bovine IAP preparation (Sigma A2356), we used a specific inhibitor of IAP, L-Phenylalanine (L-Phe)^27–29^ (Fig. 3A,B). Cells were treated with or without IAP in the presence or absence of L-Phe for 24 hrs and Baf(100 nM) was added for 3 hrs towards the end. IAP caused a significant increase in LC3-II expression when compared to the vehicle treated cells (Veh: 1.0 ± 0.17 vs IAP 25: 1.967 ± 0.2805, p < 0.05). However, in the presence of IAP25 + L- Phe, LC3-II expression was found to be comparable to the vehicle control (IAP25 + L- Phe: 0.7155 ± 0.2840, p > 0.05).To further confirm the specific effect of IAP on LC3-II expression, we also used another source of IAP, a human recombinant IAP (~10U/ml) with < 1.0 EU endotoxin per µg of the protein and with > 95% purity (Fig.S1). Similar to the bovine IAP, a pure recombinant human IAP induced autophagy confirming that the effects on autophagy were specific to IAP.

Next, we confirmed autophagy activation by IAP in cells knocked down for *Atg5*, a core autophagy gene required for the formation of autophagosomes (Fig. 3C,D). Cells were transfected with either Scrambled non-targeting siRNA (Scr) or siRNA to *Atg5*. Forty eight hrs post-transfection, cells were further treated with either the vehicle or IAP25 for 24 hrs and Baf was added for 3hrs towards the end. IAP caused a significant increase in LC3-II accumulation when compared to the vehicle control (Veh: 1.00 ± 0.30 vs IAP: 1.76 ± 0.29, p < 0.05) in cells transfected with scrambled control siRNA (Scr). However, in *siAtg5* knockdown cells, there was no significant difference between the vehicle treated and IAP treated cells (Veh: 1.00 ± 0.16 vs IAP25: 0.68 ± 0.40, p > 0.05. Taken together, our results show that IAP specifically induced autophagy in RAW cells in *Atg5*-dependent manner.

### IAP induces autophagy in a TLR4-dependent manner

**Figure 4:**
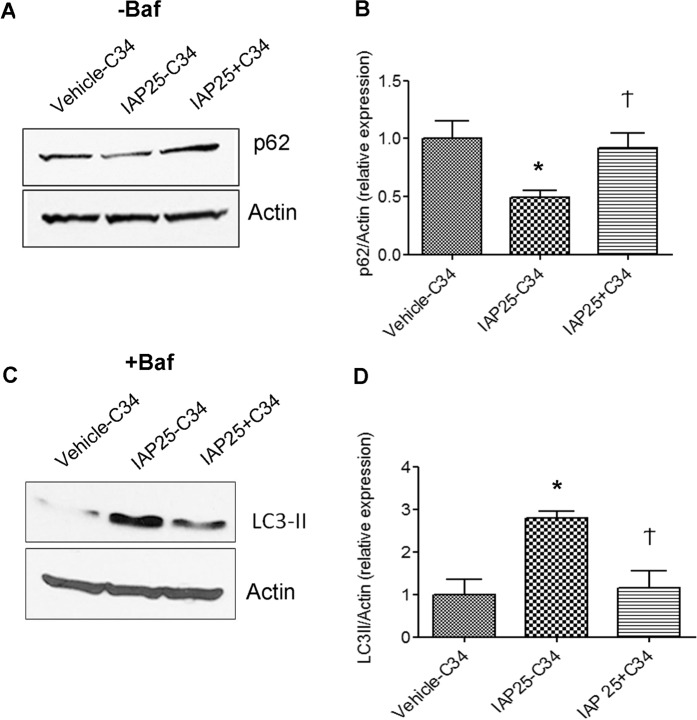
IAP-induced autophagy is dependent on TLR4 pathway. RAW cells were pre-treated without (vehicle-C34, IAP-C34) or with TLR4 INC34 (IAP25 + C34) at 15 µM for 30 mins followed by addition of vehicle or IAP (A2356) (25U/ml) for 24 hours in the absence (**A**,**B**) or presence of Baf added for 3 hrs towards the end (**C**,**D**). (**A)** Western blotting showing the expression of p62. Actin was used a loading control. The gel was cut between 100 kDa and 25 kDa (probed for p62 ~62 kDa). Following detection of p62, the blots were stripped and re-probed for Actin. (**B**) Quantification of the blots using image J (mean ± SEM from three independent experiments). (**C)** Western blotting showing the expression of LC3-II in the samples treated with Baf. Actin was used a loading control. The gel was cut between 75 kDa (probed for actin ~37 kDa) and at 25 kDa (LC3-II at ~14 kDa) (**D**) Quantification of the blots using image J (mean ± SEM from three independent experiments). One way ANOVA and Dunnett’s Multiple Comparison Test was used to determine statistical significance. *P < 0.05 and Ϯ > 0.05, compared to vehicle control.

As TLR4 activation induces autophagy^17,30^ and as IAP has been previously shown to function upstream of TLR4 signaling^15^, we asked whether IAP induced autophagy in a TLR4-dependent manner. As macrophages express abundant levels of TLR4^31^, we chose these cell lines to demonstrate the role of TLR4 in IAP-induced autophagy. RAW cells were pretreated without or with TLR4 inhibitor TLR4-IN-C34(C34) (15 µM) for 30 mins prior to adding vehicle or IAP (25U/ml) for 24 hours in the absence or presence of Baf to examine p62 and LC3-II expression, respectively (Fig. 4). Compared to vehicle treated cells, IAP treatment caused a decrease in p62 expression, a measure of autophagic degradative activity (Veh: 1.0 ± 0.27 vs IAP25: 0.4373 ± 0.04529, p < 0.05) (Fig. 4A,B). However, in the presence of TLR4 inhibitor C34, IAP failed to induce p62 degradation (0.9222 ± 0.1250, p > 0.05 compared to vehicle treated cells). Next, we treated the cells with IAP in the presence of Baf to monitor the autophagic flux (Fig. 4C,D). As observed earlier, IAP treatment led to an increase in LC3-II induction in comparison to the vehicle treatment (vehicle: 1.00 ± 0.35 vs IAP25: 2.79 ± 0.15, p < 0.01) (Fig. 4C,D). Blocking TLR4 signaling by C34 significantly inhibited LC3-II induction by IAP (1.15 ± 0.40, p > 0.05 compared to vehicle treatment) to levels comparable to vehicle treated cells. These results suggest that IAP induced autophagy in a TLR4-dependent manner. Detailed studies are needed to understand the mechanism of TLR4 signaling induced by IAP.

### IAP inhibits LPS-mediated increase in proinflammatory markers in autophagy-dependent manner

**Figure 5:**
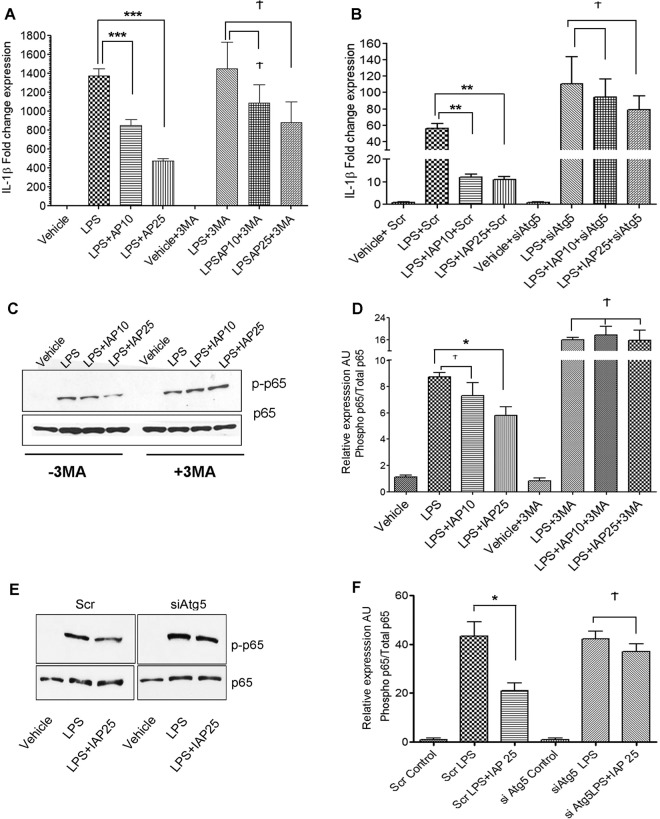
IAP inhibits LPS-mediated proinflammatory effects in autophagy-dependent manner. (**A**) RAW cells were pretreated with or without 3MA (2 mM) for 1 hour before treatment with vehicle or bovine IAP (10 and 25 U/ml) for 24 hours followed by a challenge with LPS (25 ng/ml) for 24hrs. Cells were harvested, RNA was isolated and cDNA synthesized. qPCR was carried out to determine the gene expression of IL-1β with a 2^ddCt^ method using 18 s as a housekeeping gene. Values were compared to the LPS treatment in the respective group. (**B**) Cells were transfected for 24 hrs with either scrambled siRNA or siRNA against Atg5 and treated with vehicle or IAP (A2356) at 10 or 25 U/ml for 24 hours followed by addition of LPS (25 ng/ml) for the next 24 hours. qPCR was carried out to determine the gene expression of IL-1β. Values were compared to the LPS treatment in the respective group (**C**) NF-κB activation was assessed by the phosphorylation of RelA/p65 subunit. Cells were treated with or without 3MA (2 mM) for 1 hr before adding IAP (A2356, 10 or 25 U/ml) for 24 hours followed by addition of LPS (25 ng/ml) for 15 mins. Cells were harvested and protein lysates were obtained. Western blotting was performed to detect phospho-p65. Total p65 was used as a loading control. Blot represents a gel that was cut between 100 kDa and 25 kDa probed for phospho p65 at ~65 kDa. The membrane was stripped and re-probed for total p65. (**D**) Graph represents mean ± SEM from three independent experiments). Values were compared to the LPS treatment in the respective group. (**E**) NF-κB activation was assessed in cells transfected with either scrambled siRNA or siRNA against *Atg5* and treated with vehicle or IAP (A2356) at 25 U/ml for 24 hours followed by addition of LPS(25 ng/ml) for the 15 mins. Cells were harvested and protein lysates were obtained. Western blotting was performed to detect phospho-p65. Total p65 was used as a loading control. Blot represents a gel that was cut between 100 kDa and 25 kDa probed for phospho p65 at ~65 kDa. The membrane was stripped and re-probed for total p65. (**F**) Graph represents mean ± SEM from three independent experiments. Values were compared to the LPS treatment in the respective group. One way ANOVA and Dunnett’s Multiple Comparison Test was used to determine statistical significance. *P < 0.05, ***P < 0.001 and Ϯ > 0.05.

IAP has been reported to reverse LPS -mediated proinflammatory effects^15,26,32^. We first confirmed that suppression of LPS-mediated inflammatory cascade was specific to IAP by using IAP-specific inhibitor, L-phenylalanine (L-Phe). Cells were treated with IAP in the presence or absence of L-Phe (10 mM) followed by treatment with LPS and gene expression of IL-1β was analyzed (Fig.S2A). IAP was removed from the medium before LPS was added to rule out that these effects were caused by direct binding of IAP to LPS. As expected, LPS caused a substantial increase in the fold change expression of IL-1β in comparison to the vehicle treated cells (Veh: 1.31 ± 0.52, LPS: 570.8 ± 95.17). In contrast, a significant decrease in IL-1βmRNA expression was observed in LPS + IAP treated cells when compared to LPS treatment alone (249.1 ± 10.61, p < 0.05). However, this effect of IAP was abolished in the presence of L-Phe (682.6 ± 95.33, p > 0.05 compared to LPS treatment alone) suggesting that inhibition of IL-1β expression depended on IAP. We also validated our findings using the human recombinant IAP (Fig.S2B). RAW cells were treated with recombinant IAP at 10U/ml followed by treatment with LPS. IAP containing medium was removed before the addition of LPS. LPS caused a ~200-fold increase in the expression of IL-β mRNA (231.1 ± 34.34) but this effect was inhibited in the presence of recombinant IAP (87.19 ± 24.86, p < 0.05, compared to LPS treatment). These findings validate that the anti-inflammatory effects of bovine IAP used in this study were specific to IAP. Next, we examined whether the anti-inflammatory effect of IAP against LPS was dependent on autophagy. We pretreated RAW264.7 cells with 3-methyladinine (3MA), a class III PI3K inhibitor which blocks early stages of autophagy^33^. Cells were then treated with IAP for 24 hours followed by treatment with LPS. We analyzed the relative expression of IL-1β gene in IAP versus LPS treated cells (Fig. 5A). LPS treatment increased IL-1β gene expression (1363 ± 147.3) when compared to the vehicle control cells (1.07 ± 0.24). Pre-treatment of cells with IAP caused a significant inhibition of IL-1β expression in LPS-treated cells (IAP10: 734.5 ± 101.6 and IAP25: 506.1 ± 72.12, p < 0.001). However, prior treatment with 3MA blocked the inhibitory effects of IAP and levels of IL-1β were found comparable to LPS treatment alone (LPS + 3MA: 1502 ± 296.0 vs IAP10 + LPS + 3MA: 1282 ± 364.2, and IAP25 + LPS + 3MA: 1011 ± 93.15, p > 0.05). To further confirm these findings, we knocked down *Atg5* using siRNA (Fig. 5B). We found that in cells transfected with scrambled non-targeting siRNA (Scr), IAP caused inhibition of LPS-induced upregulation of IL-1β expression (LPS + Scr: 56.02 ± 5.71 vs IAP10 + Scr: 12.03 ± 1.35 and IAP25 + Scr: 11.05 ± 1.11, p < 0.001). However, in cells knocked down for *Atg5* (*siAtg5*), IAP failed to significantly inhibit LPS-mediated increase in expression of IL-1β (LPS + *siAtg5*:110.3 ± 33.28 vs IAP10 + *siAtg5*: 94.57 ± 21.59 and IAP25 + *siAtg5*:79.03 ± 17.00, p > 0.05). These findings strongly suggest that IAP blocks LPS-mediated upregulation of IL-1β gene expression in an autophagy-dependent manner. Also, we observed that IL-1β expression was higher in LPS + siAtg5 when compared to LPS + Scr treated cells suggesting that inhibiting basal autophagy further exacerbated the LPS- mediated pro-inflammatory cascade. Disparity in the fold change expression of IL-1β in response to LPS in different experiments could be attributed to different batches and the shelf-life of LPS at the time of use.

Next, we investigated whether IAP inhibited LPS-mediated activation of NF-κB (RelA/p65) and if this effect was dependent on autophagy (Fig. 5C,D). Cells were treated with IAP or vehicle followed by treatment with LPS. To inhibit autophagy, cells were treated with 3MA before the addition of IAP. As expected, we found that LPS induced phosphorylation of p65 (p-p65), a measure of NF-κB activation. However, IAP treatment (25U/ml) significantly inhibited p65 phosphorylation when compared to LPS treatment alone (LPS: 8.72 ± 0.34 vs IAP25: 5.80 ± 0.67, p < 0.01). More interestingly, this inhibitory effect of IAP was blocked in the presence of 3MA (LPS + 3MA:15.89 ± 0.98 vs IAP25 + 3MA 15.78 ± 3.67, p > 0.05). We further validated these findings in Atg5 knockdown cells. Cells were transfected with either scrambled control siRNA or with siRNA to *Atg5*. Then, cells were treated with IAP or vehicle followed by treatment with LPS (25 ng/ml) for 15 mins (Fig. 5E,F). We found that in Scr siRNA treated cells, LPS caused an increased phosphorylation of p65 and this effect of LPS was significantly inhibited by IAP (Scr LPS: 43.40 ± 5.79 vs Scr LPS + IAP25: 21.04 ± 3.02, p < 0.05). However, in *siAtg5* transfected cells, no significant difference was observed between *siAtg5* + LPS (42.17 ± 3.14) and siAtg5 + LPS + IAP25 (37.05 ± 3.03, p > 0.05). Taken together, these findings confirm that IAP reversed LPS-mediated proinflammatory outcomes in an autophagy-dependent manner.

### IAP induces autophagy-related genes in the small intestine

**Figure 6:**
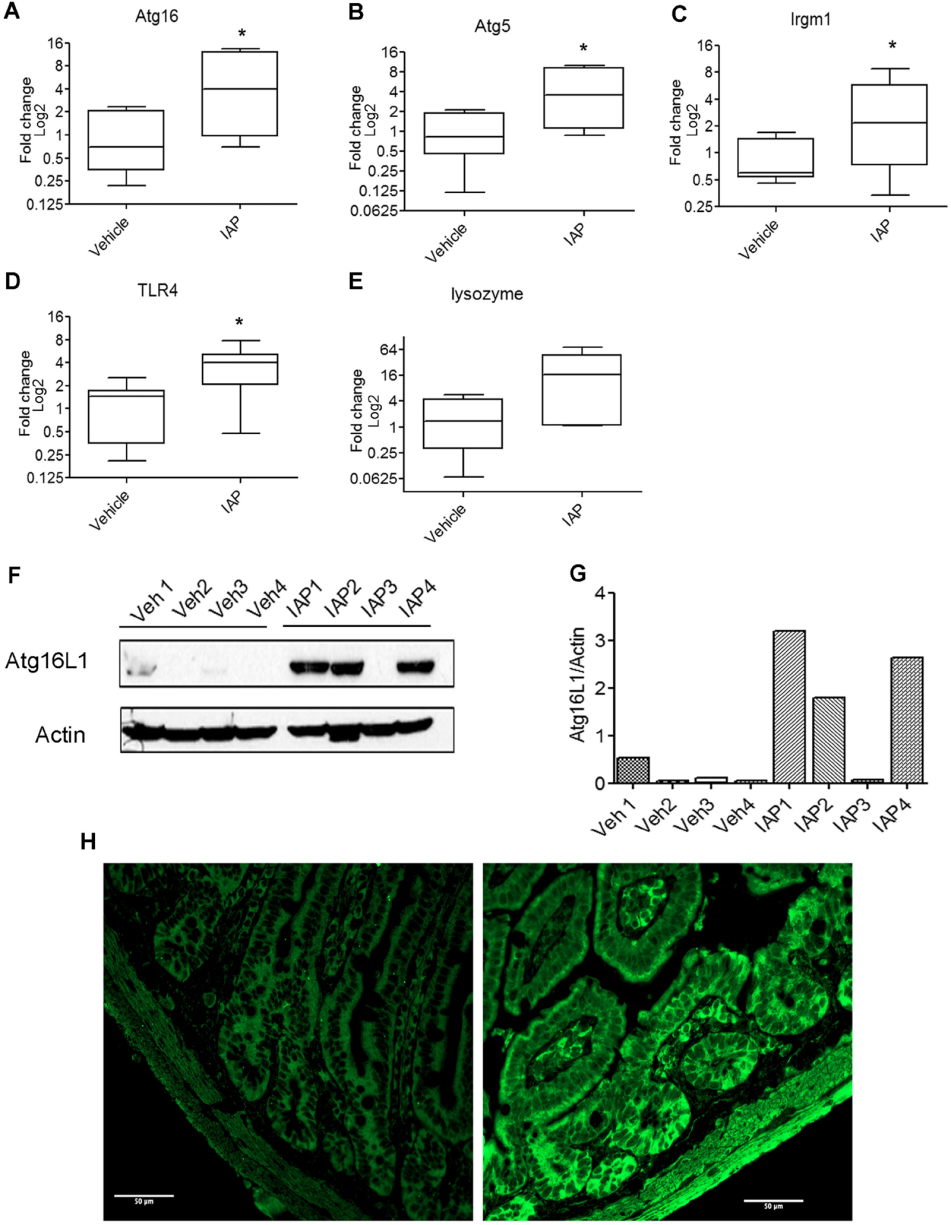
IAP induces autophagy-related gene and protein expression in the small intestine. Small intestinal ileal tissues were collected from vehicle or IAP treated animals. RNA was isolated from tissue samples and cDNA was synthesized. qPCR was carried out using primers specific for *Atg16* (**A**), *Atg5* (**B)**, *Irgm1* (**C**), *Tlr4* (**D**), and *Lyz* lysozyme (**E**) gene expression. Fold change values were calculated using 2^−ΔΔCt^ method and plotted as log base 2. Data are presented as median value with the 25^th^ and 75^th^ quartiles in each box plot. The whiskers represent highest and lowest data points. *P < 0.05. (**F**) Atg16 protein expression was analyzed in vehicle or IAP treated ileal tissues (N = 4/group) by Western blotting using anti-Atg16 antibody. Blots represent a gel that was cut at 100 kDa and at 25 kDa and probed for Atg16 ~68 kDa, followed by stripping and probing with actin antibody. (**G**) Quantification of Atg16 protein as relative expression of Atg16L1/Actin ratio. (**H**) Immunohistochemistry was performed on paraformaldehyde fixed and paraffin embedded segments of distal ileum from vehicle or IAP treated mice. Five micron sections were labeled for LC3B (Alexa Fluor 488 secondary), and imaged using a 40x oil immersion lens with wide field microscopy on an LSM 800 (Zeiss).Scale bar = 50 µm.

To investigate whether IAP induces autophagic machinery *in vivo*, we administered mice with IAP solution (100 U/ml) in the drinking water *ad libitum*. After 14 days of treatment, animals were sacrificed and ileal tissue samples were collected. We found a significant fold-change increase in *Atg16* (vehicle: 1.2 ± 0.4 vs IAP: 5.8 ± 2.0, p < 0.05) (Fig. 6A), *Atg5* (vehicle: 1.1 ± 0.3 vs IAP: 4.7 ± 1.5, p < 0.05) (Fig. 6B), and *Irgm1* (vehicle: 0.9 ± 0.2 vs IAP: 3.6 ± 1.2, p < 0.05) (Fig. 6C) gene expression in IAP treated animals when compared to the vehicle treated animals. We also analyzed the expression of *Tlr4* gene in IAP versus vehicle treated mice tissues. We found a significant increase in *Tlr4* gene expression in IAP treated animals (3.9 ± 0.9) in comparison to the vehicle treated animals (1.2 ± 0.3, p < 0.05) (Fig. 6D). These results further support that TLR4 acts downstream of IAP.

In our previous study, we showed a positive correlation between autophagy and lysozyme gene expression^34^. Thus, we analyzed the gene expression of lysozyme in animals treated with IAP. We found that there was a trend for an upregulation of lysozyme in animals treated with IAP (24 ± 10.3, p = 0.056) in comparison to the vehicle treated animals (2.1 ± 0.8) (Fig. 6E). We also analyzed Atg16L1 protein expression in vehicle vs IAP treated ileal tissue samples by Western blotting. We observed very low levels of Atg16 in mice treated with vehicle. In comparison, there was an induction of Atg16L1 in 3 out of 4 IAP treated tissues (Fig. 6F,G). We also examined the tissue expression of LC3 in the crypts of ileal tissues by immunofluorescence (Fig. 6H) and found an increase in LC3 intensity in IAP-treated tissues. Taken together, these results suggest that IAP induced core autophagy machinery genes and proteins in the ileum.

## Discussion

The role of IAP as a host defense factor in maintaining intestinal microbial homeostasis is well documented. IAP has been shown in many studies to prevent microbial dysbiosis^35–37^ and dysbiosis-related conditions such as sepsis, metabolic syndrome, colitis, and other inflammatory diseases^9,15,38,39^. The protective functions of IAP have been attributed mainly to its ability to de-toxify pro-inflammatory LPS, uridine diphosphate (UDP), adenosine triphosphate (ATP), unmethylated cytosine-guanosine dinucleotides, and flagellin, as well the regulation of bicarbonate secretion, and absorption of long chain fatty acids^3,35,36,40^. Studies have also highlighted that IAP reverses antibiotic-associated changes such as susceptibility to metabolic syndrome and susceptibility to enteric pathogens^37,41^. Malo *et al*. demonstrated that administration of exogenous IAP in mice promoted bacterial growth in stool by inactivating luminal nucleotide triphosphates^2^. Moreover, administration of IAP reduced small intestinal bacterial overgrowth in a cystic fibrosis mouse model^10^. Decreased expression of IAP has been found in inflammatory bowel disease (IBD)^7,8^ metabolic syndrome^9^, and diabetes^12^. It was found that treatment with exogenous IAP improved inflammation and gut barrier function in mouse models of these diseases^13,14^. Many inflammatory diseases often have a genetic component and may not be entirely explained by increase in LPS or in nucleotide triphosphates suggesting that IAP may also exhibit indirect protective effects via its interaction with other host factors. However, this potential aspect of IAP remains largely unexplored. A few studies have highlighted the protective role of IAP via its effect on host pathways. One such study showed that IAP maintains barrier functions by regulating the level of tight junction proteins^32^. Further, a study by Hwang *et al*. showed that IAP mediates its anti-colitis effects in a TLR-4 dependent manner uncovering yet another mechanism of IAP interaction with the host machinery. In this study, we uncovered autophagy as a novel downstream pathway activated by IAP. Autophagy has gained much attention for its protective role in the intestine in the last decade since the discovery of autophagy gene polymorphism in Crohn’s disease^42^. Many studies have since highlighted the defensive role of autophagy in the intestine in conditions such as sepsis^23^, colitis^43^, diabetes^44^, and metabolic syndrome^45^, diseases which have also been found to be associated with dysbiotic gut bacterial communities. Interestingly, the protective role of IAP has also been documented in these diseases. In this study, we found that exogenous IAP induced autophagy in human intestinal epithelial cells as well as in murine macrophage cells. We showed that IAP induced autophagy flux by using bafilomycin A1, a lysosomal inhibitor which interferes with autophagosome-lysosome fusion thereby promoting accumulation of LC3-II, and through transfection of mCherry-EGFP-LC3B expression plasmid demonstrating increase in mCherry^+^EGFP^+^ and mCherry^+^ EGFP^−^ puncta representing autophagosomes and autolysosomes, respectively. In addition, we observed a decrease in p62 expression, a selective substrate degraded by autophagy^46,47^. We also found that induction of autophagy by IAP was dependent on signaling through TLR4, a pattern recognition receptor, as IAP failed to activate autophagy in the presence of TLR4 inhibitor C34. IAP was previously found to inhibit LPS-induced TNFα and IL-6 proinflammatory cytokine production, NF-κB DNA-binding activity, LPS-induced IκBα phosphorylation /degradation, and to reduce the severity of DSS-induced colitis in mice in a TLR4-dependent manner^15^. As TLR4 is a sensor for autophagy, our results provide a mechanism by which IAP may induce autophagy via TLR4 signaling. It remains to be determined how IAP may activate TLR4 signaling. IAP and TLR4 have been shown to colocalize in Crohn’s disease patients and in healthy control subjects^48^. Our animal data also showed that IAP treatment caused an increase in TLR4 gene expression in the ileum. This result is supported by Hwang *et*. *al* who showed that IAP protected against DSS-induced colitis in a TLR4- dependent manner thus suggesting that IAP activates the TLR4 pathway to mediate its effects^15^. However, the mechanism of how IAP may activate and increase TLR expression remains elusive. TLR4 pathway has been shown to be activated by not only bacterial ligands such as LPS but also by endogenous ligands such as fibronectin and heat shock proteins^49,50^. It is possible that IAP activates TLR4 in a similar manner. IAP may also bind to TLR4 or to its co-receptor molecules CD14 and MD2. Increased TRL4 expression and its activation by IAP may also enhance sensitivity of the intestinal tissue to potential microbial challenge by activating intracellular autophagy. Demonstration of a possible direct interaction between IAP and TLR4 would be valuable in understanding how IAP may activate TLR4. IAP interaction with TLR4 and the activation of downstream cascade remains the subject of future research.

We also show that IAP inhibited LPS -mediated increase in IL-1β expression as well as LPS-mediated activation of NF-κB (p65 subunit) and these effects of IAP were blocked by either 3MA or Atg5 knockdown. The anti-inflammatory role of IAP against LPS has been demonstrated in a few studies^15,26,32^. In a study by Hamarneh *et al*. IAP supplementation was found to decrease IL-1β mRNA expression in ileal tissue in alcohol-induced mouse model of intestinal inflammation^51^. Interestingly, Hwang *et al*. suggested that IAP exerted anti-inflammatory effects may be mediated by another anti-inflammatory mechanism rather than direct detoxification of LPS as pre-incubation of cells with IAP causes a stronger inhibition of cytokine secretion when compared to the cells treated with an LPS and IAP pre-mixture. Our findings allude to autophagy as that other anti-inflammatory mechanism induced by IAP, acting downstream of TLR4 activation and upstream of NF-κB signaling. While autophagy has been shown to inhibit LPS-mediated IL-1β production mainly by inactivating inflammasomes^52^, a few studies suggest that autophagy may also affect the transcription of IL-1β. It was reported in PBMCs derived from CD patients with Atg16 risk allele that the expression of IL-1β mRNA in response to MDP was higher than that of controls^53^. In other studies, induction of autophagy by starvation inhibited IL-1β transcription while 3MA treatment reversed this effect and caused an increase in IL-1β mRNA levels in human PBMCs^54,55^. While the effect of autophagy on IL-1β transcription appears to be the property of human monocytes, the effect of autophagy on IL-1β at the transcriptional level in mice has also been reported. It was observed that autophagy induction by starvation caused a decrease in IL-1β mRNA expression in RAW cells^56^. In another study, inhibition of autophagy by 3MA was found to cause an upregulation of IL-1β mRNA expression in adipose tissue in obese mice^57^. Our finding that IAP-induced autophagy inhibited LPS-mediated upregulation of IL-1β mRNA and that this effect was suppressed by 3MA or by *Atg5* siRNA concurs with these studies. Whether or not IAP-induced autophagy inactivates inflammosome to affect IL-1β production remains to be determined.

We also tested the effect of IAP on autophagic machinery in the ileum in mice. We detected an upregulation of autophagy genes *Atg5*, *Atg16*, and *Irgm* in response to IAP which further confirmed our findings *in vitro*. We did not demonstrate a functional autophagy such as increase in LC3-II expression in animal tissues treated with IAP due to technical difficulties in measuring autophagy flux in the animal tissues at the time point tested. However, many studies have reported that increase in gene and protein expression of the autophagy pathway is concurrent with the induction of autophagy^58,59^. However, we did observe an increase in protein expression of Atg16L1 in IAP treated tissues which supported our qPCR data. IAP was also found to cause upregulation of *Tlr4* expression which is in agreement with the previous study showing that IAP acts upstream of TLR4^15^. In addition, we also found that IAP treatment induced *Lyz* expression in the ileum. Lysozyme and other AMPs are innate antibiotics that not only eliminate pathogens but also shape the resident gut microbial community owing to their antimicrobial properties^60^. Several studies have reported that IAP and lysozyme expression show the same trend in the one direction in response to various stimuli under experimental conditions^60–63^. To our knowledge, this is the first study that demonstrates a positive downstream effect of IAP on lysozyme expression. As autophagy is a positive regulator of lysozyme^64^, it is possible that autophagy activation in the gut by IAP may lead to activation of lysozyme and possibly other AMPs which are secreted in the intestinal lumen. As IAP is known to regulate gut microbes, it will be interesting to study whether IAP-induced autophagy contributes to anti-microbial effects of IAP by activating AMPs. This putative mechanism would amplify the antimicrobial cascade triggered by IAP via autophagy thereby efficiently orchestrating the host defense mechanisms to control bacterial overgrowth in the intestine especially in the regions where IAP activity is low and where direct antimicrobial effects of IAP may be inadequate. Further studies are needed to confirm these findings.

Based on our overall observations, we suggest a mechanism whereby intestinal IAP induces autophagy via a TLR4-dependent pathway which inhibits the LPS-mediated pro-inflammatory effects. In conclusion, our findings identify IAP, an intestinal host defense protein, as a novel physiological inducer of autophagy and demonstrate that IAP-induced autophagy inhibits the transcription of IL-1βmRNA in the presence of LPS by affecting NFκB activation. This study advances our understanding of how IAP may function to limit inflammation in the setting of dysbiosis by not only directly detoxifying LPS but also by signaling the host autophagy to activate downstream pathways in a two-prong approach. Our study also provides a framework for using IAP to induce autophagy *in vitro* and in various animal models of dysbiosis and lays the foundation for further studies into the role of IAP for conditions that are associated with dysfunctional autophagy.

## Materials and Methods

### Animals

Five-week-old female C57BL/6 mice (20–25 g) were utilized in this study. Mice were purchased from Charles River Laboratory (Wilmington, DE). Upon arrival, animals were housed in groups of 4 in polypropylene cages and placed on a 12-hour light/dark cycle and kept on a standard rodent diet (Harlan Teklad Laboratory Diets). Mice were subjected to one-week acclimatization period.

### Study approval

Procedures were approved by the Institutional Animal Care and Use Committee at the New Mexico VA Health Care System following guidelines provided by the Guide for the Care and Use of Animals of the National Research Council.

### Cell culture and treatments

HCT116 and RAW 264.7 cells were purchased from ATCC (Manassas, VA). RAW cells were grown in DMEM supplemented with 10% fetal bovine serum and HCT116 were grown in McCoys 5 medium supplemented with 10% fetal bovine serum. No antibiotics were added to the culture media for any of the cell lines. Cells were grown at 37 °C in a humidified incubator with 5% CO_2_. Cells were grown to ~70% confluency before treatment for 24 hours with various concentrations of bovine intestinal alkaline phosphatase (Sigma: A2356) in the absence or presence of bafilomycin A1, an autophagy flux inhibitor (100 nM, added for 3 hrs towards the end of 24 hr incubation.) (Sigma: SML1661). Vehicle treated cells were used as negative controls (the volume of vehicle and IAP was adjusted to 2 µl/well in a 6-well plate). Results with bovine IAP were validated with the use of recombinant human IAP (13225-H08H, Sino Biological) (Accession:P09923) without the pro-peptide and with a c-terminal His-tag and by using L-phenylalanine (L-Phe), a specific inhibitor of IAP. TLR4 inhibitor TLR4-IN-C34 (Sigma: SML0832) was added to RAW cells at a concentration of 15 µM for 30 mins before the addition of IAP for 24 hours. LPS (Sigma: L4391) was added to the cells at 25 ng/ml for 15 mins or 24 hrs. 3-Methyladenine (3MA) (Sigma; M9281) was added at 2 mM for 1 hr prior to the addition of IAP. L-phenylalanine (L-Phe) was added as an inhibitor of IAP at the concentration of 10 mM. Cells were harvested and processed for the preparation of protein and RNA samples.

### Western blot

Cells and tissues were lysed in Lysis buffer (ThermoFisher: 87787) containing protease and phosphatase inhibitors (ThermoFisher: 1861281). Protein concentration was determined with a Bradford reagent (Bio-Rad). Fifty µg proteins from each sample were run on SDS-PAGE and transferred to nitrocellulose membranes. Membranes were blocked in 5% milk in PBS-T (0.1%Tween 20) for 30 mins followed by overnight incubation in cold in antibodies against Actin (Cell Signaling: 4970), p62 (Cell Signaling: 5114) or LC3 (Cell Signaling: 3868), Atg16 (Cell Signaling: 8089), p65 and phospho-p65 (Cell Signaling: 4764 and 3033, respectively). Antibodies were diluted 1:1000 in 5% BSA in PBS-T as recommended by the manufacturer. Blots were incubated with secondary antibodies (Cell Signaling: 7074) at room temperature for 1 hour (dilution of 1:2000) and developed using enhanced Chemiluminiscence signal (ThermoFisher: 32106).

### Plasmid transfection

HCT116 cells were transfected with pBABE-puro mCherry-EGFP-LC3B plasmid for 24 hours using lipofectamine 2000 reagent (ThermoFisher). The plasmid was kindly provided by Dr. Xiang Xue at the University of New Mexico. RAW cells were transfected with the same plasmid using a nucleofector kit V (Lonza). After 24 hours, cells were treated with IAP at various concentrations for 24 hours. Vehicle treated cells were used as controls. Cells were washed twice with PBS and fixed with 4% paraformaldehyde for 15 mins at room temperature. Cells were then washed 3 times with PBS and mounted with permaflour mounting medium (ThermoFisher). Cells were observed and imaged with Olympus FluoView FV1200 confocal microscope.

### siRNA transfection

Scrambled siRNA (scr) and siRNA against mouse *Atg5* were purchased from Dharmacon. Thirty five nM siRNA was used for transfection of RAW cells using lipofectamine 2000 (ThermoFisher Scientific: 52758) using manufacturer’s protocol. At 24 hours post- transfection, cells were removed and plated into 6-well plates. After an additional 24 hours, cells were treated with the IAP or vehicle for 24 hrs followed by addition of LPS (25 ng/ml) for the next 24 hrs. IAP was removed from the medium prior to adding LPS.

### IAP treatment in mice

Mice (N = 8 per group) were administered either IAP (Sigma: A2356)(100 U/ml) or equal volume of vehicle (50% glycerol containing 5 mM Tris, 5 mM MgCl_2_, and 0.1 mM ZnCl_2_, pH 7.0) in their drinking water for 14 days with *ad libitum* access. On day 15, mice were sacrificed and small intestinal tissues were collected and stored in RNA later at −80 °C for further analysis.

### Immunohistochemistry

Vehicle or IAP treated ileal tissues were fixed in 4% paraformaldehyde and then processed and embedded in paraffin for sectioning. Sections (5 mm) were cut from each embedded tissue onto positively charged slides. Slides were deparaffinized with subsequent xylene and ethanol washes and then rinsed in distilled water. For antigen retrieval, Sodium Citrate Buffer (10 mM Sodium Citrate, 0.05% Tween 20, pH 6.0) was heated to approximately 95 °C in a staining dish. Once temperature was reached, slides were immersed in the staining dish and incubated for 30 minutes at temperature. The staining dish was then removed to room temperature and allowed to cool. Slides were then rinsed twice with PBS with 0.05% Tween 20 (PBST) for 2 min each. Sections were blocked for 30 minutes with Fetal Bovine Serum. They were then incubated with antibodies in dilution buffer (PBST with 0.5% FBS), 1:250 of anti-LC3B (Cell Signaling), and 1:500 anti-rabbit Alexa Fluor 488 (Cell Signaling) overnight at 4 °C. In between the incubations, sections were washed three times with PBST for 5 min. After last wash, excess moisture was removed from sections and coverslips were affixed using ProLong Gold (ThermoFisher). Wide-field fluorescence imaging of gut tissue sections was performed utilizing an automated tiling application (Zen2.3, Carl Zeiss Microscopy) with images acquired with a 40 × (NA 1.3), oil immersion Plan Apochromat objective on an inverted AxioScope A.1 equipped with an AxioCam506 monochrome camera. Images were equivalently brightness/contrast enhanced.

### Quantitative PCR

RNA was extracted from intestinal tissues (RNeasy Mini Kit, Qiagen: 74106). The cDNA synthesis was carried out using cDNA kit (ThermoFisher: 18080–051). Real–time quantitative PCR was performed on Applied Biosystems QuantStudio 7 Flex (ThermoFisher) using SYBR green (Qiagen: 204145). Host gene expression was analyzed by calculating fold change values using 2-^ddct^ method. Gene expression of 18s  was used as a housekeeping control. The primer sequences are described below: qPCR conditions were as follows (Table 1):

**Table 1:** Primer Sequences.

18s F	GTAACCCGTTGAACCCCAT
18s R	CCATCCAATCGGTAGTAGCG
*Lyz* F	GGATCAATTGCAGTGCTCTG
*Lyz* R	CAGTTCCGAATATACTGGGAC
*Atg5* F	ATATGAAGGCACACCCCTGA
*Atg5* R	CCAAGGAAGAGCTGAACTTGATG
*Atg16* F	AGGCGTTCGAGGAGATCATT
*Atg16* R	TTCTGCTTGTAGTTTCTGGGTCA
*TLR4* F	GGTTGAGAAGTCCCTGCTG
*TLR4* R	ATTCGAGGCTTTTCCAT
*Irgm1* F	GAGACTGTGGCAACATTG
*Irgm1* R	CCGATGACTCGAAGTGCATTG
*IL-1β* F	TGAAATGCCACCTTTTGACAG
*IL-1β* R	CCACAGCCACAATGAGTGATAC



### Statistical analysis

All graphs were generated using Graph Pad Prism 5. For data analysis, we compared differences between two groups using a two-tailed t-test. One-way ANOVA with a post-hoc Dunnett’s multiple comparison test was used for comparing the difference between three or more groups. P values < 0.05 were considered significant. All *in vitro* experiments were carried out at least three independent times (N = 3). Statistical analysis was carried out with the help of an onsite statistician Clifford Qualls, PhD.

## Supplementary information

Supplementary information
