# Supplementary material for: Intestinal Alkaline Phosphatase Exerts Anti-Inflammatory Effects Against Lipopolysaccharide by Inducing Autophagy
Source: Sci Rep. 2020 Feb 20;10:3107. doi: 10.1038/s41598-020-59474-6 (PMC7033233; doi:10.1038/s41598-020-59474-6)
Supplement: Supplementary file 1 — Supplementary information [file 41598_2020_59474_MOESM1_ESM.docx]

**Intestinal Alkaline Phosphatase Exerts Anti-Inflammatory Effects**

**Against Lipopolysaccharide by Inducing Autophagy**

Sudha B. Singh^1^, Amanda Carroll-Portillo^1^, Cristina Coffman^1^, Nathaniel L. Ritz^1*^,

and Henry C. Lin^2,3#^

**Figure S1.** RAW264.7 cells were treated with recombinant IAP (13225-H08H, Sino Biological) for 24 hrs and Baf (100nM) was added towards the end for 3 hours. LC3 II induction was analyzed by western blotting and images were quantified using Image J software. Dotted line represents a cut in the gel where upper portion (between 75kDa and 25kDa) was probed for Actin and the lower portion (below 25kDa) was probed for LC3.

**Figure S2. (A)** RAW264.7cells were treated with vehicle or IAP (Sigma:A2356) at 25U/ml for 24 hours in the presence or absence of L-Phe (10mM) followed by treatment with LPS (25ng/ml) for 24 hours. IAP containing medium was removed before the addition of LPS. Cells were harvested, RNA was isolated, and cDNA synthesized. QPCR was carried out to determine the gene expression of IL-1β. Relative fold change expression was calculated using 2^-ddct^ method using 18S gene expression as a housekeeping control. Values represent mean±SEM from three independent experiments and were compared to LPS. One way ANOVA and Dunnett’s Multiple Comparison Test was used to determine statistical significance. *P <0.05 and Ϯ P >0.05. (**B**) RAW cells were treated with or without recombinant IAP (13225-H08H, Sino Biological) at 10U/ml for 24 hours followed by incubation with LPS for 24 hours. IAP containing medium was removed before the addition of LPS. Bar graph represents qPCR analysis of IL-1β mRNA expression. Student’s t-test as used to compare the difference between the LPS group and IAP+ LPS. Values represent mean±SEM from three independent experiments and were compared to LPS. *P <0.05.**Original Blots for the main and supplemental figures**

z


Fig. 2A

Cut at 100

kDa

Cut at 25

kDa

Actin

p62

Stripped and probed for Actin

Fig.3A


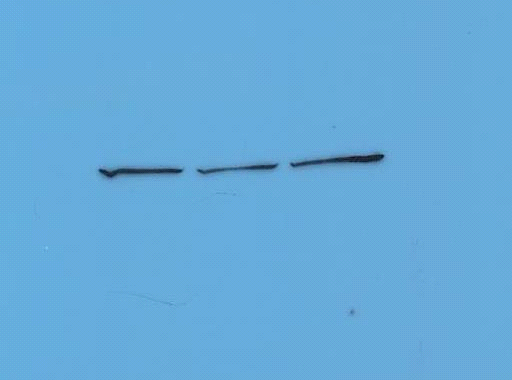

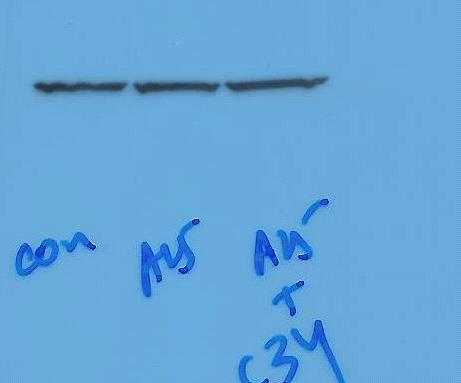


Fig. 4A
